# Bioassay-Guided Fractionation Networking for Discovery of Biofungicides from Cultivated *Salvia canariensis*

**DOI:** 10.3390/ijms252413323

**Published:** 2024-12-12

**Authors:** Eduardo Hernández-Álvarez, Samuel Rodríguez-Sabina, Guacimara González-Delgado, Carolina P. Reyes, Cristina Giménez, María Ángeles Llaría-López, Raimundo Cabrera, Isabel L. Bazzocchi, Ignacio A. Jiménez

**Affiliations:** 1 Instituto Universitario de Bio-Orgánica Antonio González, Departamento de Química Orgánica, Universidad de La Laguna, Avenida Astrofísico Francisco Sánchez 2, 38206 La Laguna, Tenerife, Spain; alu0100947311@ull.edu.es (E.H.-Á.); ilopez@ull.edu.es (I.L.B.); 2 Departamento de Botánica, Ecología y Fisiología Vegetal, Universidad de La Laguna, Avenida Astrofísico Francisco Sánchez, 38206 La Laguna, Tenerife, Spain; srodrisa@ull.edu.es (S.R.-S.); cgmarino@ull.edu.es (C.G.); rcabrera@ull.edu.es (R.C.); 3 Área de Gestión del Medio Natural y Seguridad, Cabildo Insular de Tenerife, C/ Las Macetas s/n, Pabellón Insular Santiago Martín, 38108 La Laguna, Tenerife, Spain; guacimaragd@tenerife.es (G.G.-D.); mllaria@tenerife.es (M.Á.L.-L.); 4 Instituto Universitario de Bio-Orgánica Antonio González, Departamento de Bioquímica, Microbiología, Biología Celular y Genética, Universidad de La Laguna, Avenida Astrofísico Francisco Sánchez 2, 38206 La Laguna, Tenerife, Spain; cpreyes@ull.edu.es

**Keywords:** *Salvia canariensis*, plant cultivation, biofungicide-guided fractionation, abietane-type diterpenoids, pest control

## Abstract

Considering the detrimental impacts of the current pesticides on the biotic components of the biosphere, the development of novel pesticides is vital. Plant-derived biopesticides have emerged as popular alternatives to create a safer and more sustainable agriculture model. This study aims to validate the previous bioguided fractionation of endemic Canary Islands sage, *Salvia canariensis*, as a potential source of botanical pesticides using a cultivation process. Accordingly, the bioassay-guided fractionation of the ethanolic extract of the leaves of cultivated *S. canariensis* on the phytopathogenic fungal mycelia of *Botrytis cinerea*, *Fusarium oxysporum*, and *Alternaria alternata* yielded six known terpenoids. Their abietane diterpenoid-type (**1**–**5**) and sesquiterpenoid (**6**) structures were established based on spectroscopic and spectrometric analysis. This strategy identified one abietane diterpenoid, salviol (**5**), as a potential candidate for the future development of biofungicides with similar potency towards the assayed phytopathogenic fungi to commercial fungicides. Salviol worked in a concentration-dependent manner. Overall, this study reinforces the potential of abietane-type diterpenoids as promising agrochemical lead compounds against infectious diseases caused by phytopathogenic fungi and validates the cultivation of *S. canariensis* as a potential source of plant-derived biopesticides.

## 1. Introduction

The increasing global population and food demand [1] and the spread of plant diseases are regarded as serious challenges to global food security [2]. Crop losses caused by phytopathogenic fungi have a devastating impact [3], especially those species from the *Alternaria* [4], *Botrytis* [5], and *Fusarium* [6] genera. For over a century, synthetic pesticides, also known as agrochemicals, have been primarily used for the management of plant pests and diseases. However, their widespread and unregulated use has resulted in risks to human health and the environment [7].

Therefore, the development of effective and less harmful fungicides is expected to have an impact on plant diseases and food security. Recently, the search for eco-friendly products has focused on biological pesticides, botanical biopesticides, and plant-oriented chemicals, because they are non-hazardous, sustainable, and effective alternatives against phytopathogens [8]. Thus, structural diversity studies of natural products could play a notable role in the development of biopesticides for integrated pest control. In fact, some secondary metabolites, such as terpenoids, flavonoids, alkaloids, and essential oils, have already been reported as promising in pest control [9]. Even so, the potential use of natural products for eco-friendly solutions to crop protection has not been satisfactorily explored [10].

*Salvia*, with nearly 1000 species and a wide-ranging distribution, is one of the largest genera in the Labiatae (*Lamiaceae*) family [11]. The economic importance of the *Salvia* species continues to increase based on modern healthy lifestyle claims of its suitability as a quality food and nutraceutical supplement of natural origin. Nowadays, because of its valuable medical, food flavoring, and preservative properties, many *Salvia* species have been widely used as ingredients in the food, pharmaceutical, and cosmetic industries [12]. Thus, the growing demand for *Salvia* plants has led to the overexploitation of its natural habitats; therefore, there is an urgent need for a sustainable source of *Salvia* biomass to prevent an ecological crisis [13]. *Salvia* species biosynthesize a wide variety of bioactive secondary metabolites, including diterpenoids, phenolic acids, triterpenoids, and flavonoids. Diterpenoids from this genus belong almost invariably to the abietane-type skeleton, and they exhibit a wide range of biological activities, which have increased the interest in *Salvia* species among the agricultural, medicinal, and pharmacological communities [14,15,16]. Previous phytochemical analyses on wild-growing *Salvia canariensis*, an endemic plant from the Macaronesian region, have reported the isolation of abietane-type diterpenoids with remarkable growth inhibition in phytopathogenic fungi [17].

However, the discovery of biopesticides from a plant species can lead to the overexploitation of natural habitats. Therefore, the increased cultivation of the target species is required as a sustainable strategy [18] to ensure the availability of biomass. To continue our efforts towards the discovery of novel plant-derived biopesticides for sustainable agriculture, the present study aims to cultivate and validate previous results on wild *S. canariensis* as a promising source of biopesticides. Herein, we report the isolation and structural identification of five known abietane-type diterpenoids (**1**–**5**) and one sesquiterpenoid (**6**) from the ethanolic extract of the leaves of cultivated *S. canariensis* using bioassay-guided fractionation performed against the phytopathogens *Alternaria alternata*, *Botrytis cinerea,* and *Fusarium oxysporum*, which are fungal diseases affecting crops worldwide. The isolated diterpenoids showed remarkable antifungal activity. A phytochemical comparative analysis between cultivated and wild-type *S. canariensis* is also presented.

## 2. Results and Discussion

### 2.1. Bioassay-Guided Fractionation

**Figure 1:**
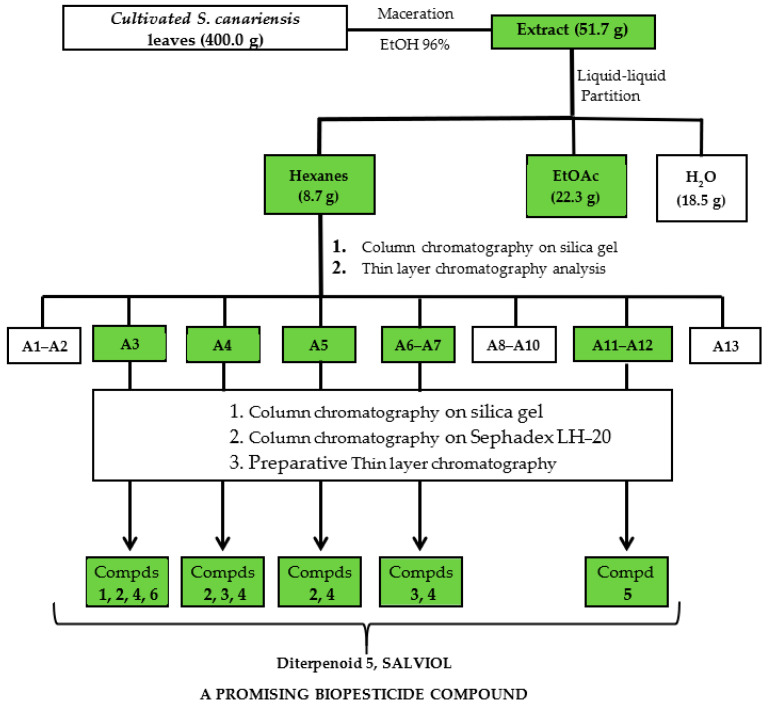
Bioactive natural product discovery pipeline. Flowchart of bioassay-guided fractionation of cultivated *S. canariensis* against *F. oxysporum*, *B. cinerea*, and *A. alternata*.

The goal of the current work was the development and validation of cultivated *Salvia canariensis* as a potential source of biopesticide platforms through bioguided fractionation (Figure 1). The growth inhibition percentage (%GI) was determined by a test at the mycelium stage [19] in three phytopathogenic fungi: *Botrytis cinerea*, *Fusarium oxysporum*, and *Alternaria alternata*. Commercial antifungal agents Fosbel-Plus (35% Fosetil Al and 35% Mancozeb) and Ortiva PC (Azoxystrobin 250 g/L, 22.8% p/p) were used as positive controls, whereas ethanol was used as a negative control.

Initially, the ethanolic extract of the leaves obtained by maceration (12.9% yield, plant dry mass) was evaluated for its antifungal activity (Table 1). The results showed remarkable growth inhibition activity at a concentration of 1 mg/mL, with percentages of growth inhibition of 68.6%, 41.1%, and 53.6% against *A. alternata*, *B. cinerea*, and *F. oxysporum*, respectively; even at 0.1 mg/mL, it showed some degree of activity (45.0, 29. 5, and 21.3%, respectively). Moreover, these data demonstrated that the ethanolic extract exhibited fungicidal activity in a concentration-dependent manner. These results are similar to those for the wild-type plant [17], indicating that cultivated *S. canariensis* is a promising source of plant-derived biopesticides. Additionally, the fungicidal activity was only slightly lower than that of the positive control, highlighting the potential to use the plant extract directly as a botanical pesticide for crop protection against these phytopathogenic fungi. These results show that the phyto-extract confirms that the plant can be cultivated without loss of fungal activity. Such a cultivation strategy would ensure the availability of sufficient biomass at the lowest possible environmental cost, relieving the pressure on the natural ecosystem.

Subsequently, the EtOH extract was fractionated by liquid–liquid partition. Then, the dry mass was suspended in distilled water (H_2_O) and extracted using solvents of increasing polarity, namely hexane (Hx, 2.2% yield, plant dry mass) and ethyl acetate (EtOAc, 5.6% yield, plant dry mass), to obtain the enriched fractions. The bioactivity from this first separation step revealed that the organic fractions, the Hx (%GI ranging from 52.4 to 73.5) and EtOAc (%GI ranging from 19.8 to 59.3) fractions, were more active than the aqueous fraction (ranging from not active to 46.7%GI) at 1 mg/mL. The extract and fractions with growth inhibition higher than 20% at 1 mg/mL were assayed at lower concentrations (0.5 and 0.1 mg/mL) (Table 1 and Table S1 in the Supplementary Materials). The fungicidal activity from the liquid–liquid partition showed that the active components presented polarity that ranged from medium to low, which could be useful to optimize the extraction and to develop effective and selective methods in the future.

**Figure 2:**
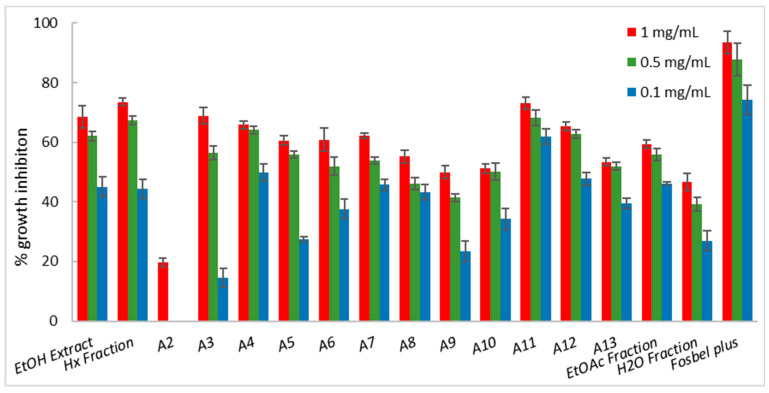
Antifungal effects (% growth inhibition) of plant extract, fractions, and sub-fractions (A2–A13) against *Alternaria alternata*.

**Figure 3:**
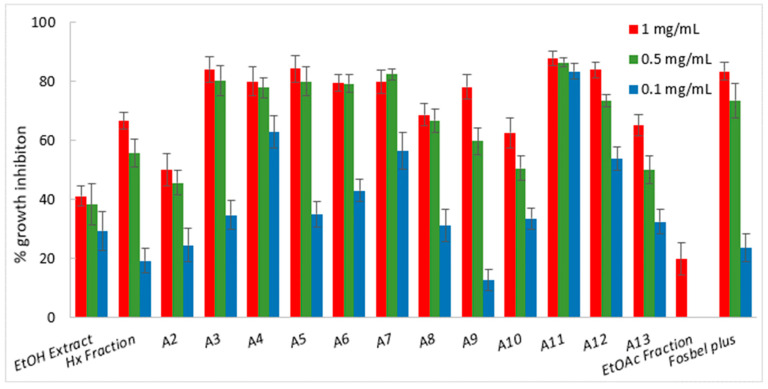
Antifungal effects (% growth inhibition) of plant extract, fractions, and sub-fractions (A2–A13) against *Botrytis cinerea*.

**Figure 4:**
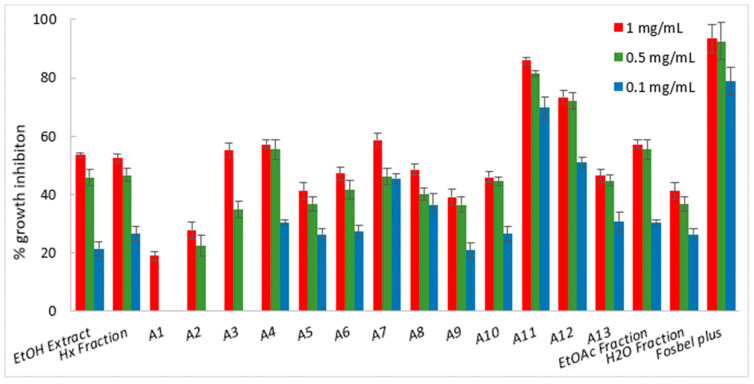
Antifungal effects (% growth inhibition) of plant extract, fractions, and sub-fractions (A2–A13) against *Fusarium oxysporum*.

Among the fractions from the liquid–liquid partition, the Hx fraction showed the best fungicide profile, exhibiting higher or similar %GI values at 1 mg/mL than the ethanolic extract, suggesting that this fraction was enriched in bioactive components. Consequently, the Hx fraction was selected for further fractionation by column chromatography on silica gel and in thin-layer chromatographic analyses, affording thirteen sub-fractions (A1–A13), which were screened for their antifungal profiles (Figure 2, Figure 3 and Figure 4). This chromatographic stage led to an increase in the fungal growth inhibition rate, with a decrease in concentration from the Hx fraction at 1 mg/mL to the sub-fractions at 0.5 mg/mL (Table S1 in the Supplementary Materials).

Among these sub-fractions, seven (A3–A7, A11, and A12) showed remarkable activity, exhibiting growth inhibition of up 60% against *A. alternata*. Moreover, sub-fraction A11 showed a %GI of 61.9 versus the Hx fraction with a %GI of 44.3 at 0.1 mg/mL, resulting in a 1.4-fold increase in activity (Figure 2).

Regarding the results for *B. cinerea*, almost all sub-fractions (A2 to A12) showed an improvement in potency in comparison with the Hx fraction at 1 mg/mL (Figure 3). In fact, sub-fractions A4 and A11 exhibited notable activity (%GI 62.8 and 83.4, respectively) at the lower assayed concentration of 0.1 mg/mL, being 3.3 and 4.3-fold, respectively, more active than the Hx fraction (%GI 19.3).

Likewise, five sub-fractions (A3, A4, A7, A11, and A12) displayed growth inhibition of up to 50% against *F. oxysporum* at 1 mg/mL (Figure 4). Moreover, sub-fractions A11 and 12 (%GI 69.7 and 51.2, respectively) at 0.1 mg/mL resulted in an increase in potency of 2.6- and 1.9-fold with respect to the Hx fraction. In this sense, sub-fraction A11 possessed the best fungicide profile, with %GI values > 70% against the three phytopathogenic fungi. This was similar to that of the positive control at all assayed concentrations.

**Figure 5:**
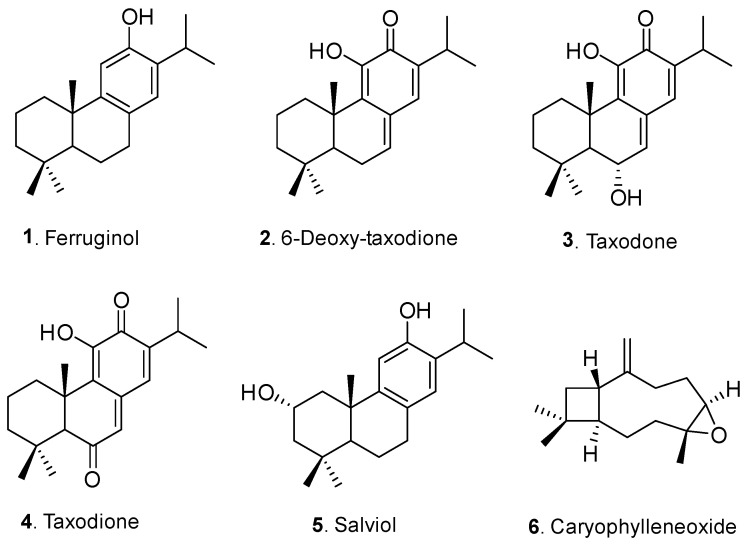
Chemical structures of isolated compounds (**1**–**6**) from cultivated *S. canariensis*.

The most active subfractions, A3–A7 and A11–A12, were subjected to several purification steps by column chromatography and preparative TLC to obtain the isolated compounds **1**–**6** (Figure 5), as described in detail in the Supplementary Materials (Figures S1–S4). The characterization of each isolated terpenoid was carried out via the extensive analysis of their NMR spectroscopic data and the comparison of their spectral data with those reported in the literature. The isolated compounds were identified as ferruginol (**1**) [20], 11-hydroxy-12-oxo-7,9 (11)-13-abietatriene (6-deoxy-taxodione, **2**) [21], taxodone (**3**) [22], taxodione (**4**) [23], salviol (**5**) [24], and caryophyllene oxide (**6**) [25].

### 2.2. Antifungal Activity Assays of the Isolated Compounds

The effect on the fungal viability of the six compounds (**1**–**6**) isolated from the bioguided assay was evaluated individually against the three phytopathogenic fungi (Table 2), Compounds with growth inhibition higher than 20% at 0.1 mg/mL were assayed at lower concentrations (0.05 and 0.01 mg/mL).

The results of the in vitro assays against *A. alternata* revealed that four diterpenoids (**1**–**4**) showed moderate fungicidal activity, with %GI values ranging from 35.3 to 40.9 at 0.1 mg/mL. Furthermore, diterpenoid **5** (salviol) exhibited the best inhibition effect (%GI 72.3), with similar levels to the positive control (Fosbel-Plus, %GI 74.2). The evaluation on *B. cinerea* revealed that diterpenoids **1**–**5** showed moderate to potent activity, with compound **5** (%GI 81.2) exhibiting 1.2- and 3.4-fold more activity than the phytosanitary products, i.e., azoxytrobin and Fosbel-Plus, respectively, at 0.1 mg/mL. Furthermore, the antifungal activity against *F. oxysporum* indicated that compound **5** (%GI 74.1) showed similar growth inhibition to the positive control (Fosbel-Plus, %GI 78.8), whereas the other diterpenoids exhibited low or moderate antifungal activity (%GI 23.5 to 33.7) at 0.1 mg/mL. Based on these results, the EC_50_ values of the most active compound **5** were determined against the three phytopathogenic fungi, exhibiting values of EC_50_ of 0.015 mg/mL for *A. alternata* and *B. cinerea* and 0.089 mg/mL for *F. oxysporum*. Moreover, compound **5** was more active against *A. alternata* than the positive control, Fosbel-Plus (EC_50_ 0.020 mg/mL). No noteworthy antifungal activity was detected against the tested fungi for caryophyllene oxide (**6**) at the higher evaluated concentration (0.1 mg/mL), with the %GI values being lower than 10% for *B. cinerea* and *F. oxysporum* and 12% against *A. alternata*. The potent activity shown by salviol supports previous works’ claims [26,27] that natural product derivatives could be promising candidates and eco-friendly alternatives that could reduce the use of chemical fungicides, as they are effective in the micro- to millimolar range.

Despite the great deal of research conducted on the biological properties of abietane diterpenoids, there are few reports available on their biopesticide potential. In a previous work, Kofujita et al. [28] reported the antifungal activity of ferruginol against the phytopathogenic fungi *Alternaria alternata*, *Pyricularia oryzae*, *Rhizoctonia solani*, and *Fusarium oxysporum*, showing relatively high activity; however, the fungal strains and methodology used were different to those reported herein, prohibiting a comparative analysis. Moreover, ferruginol was found to exhibit excellent antifungal activity against the brown root rot fungus *Phellinus noxius*, a fungus that causes severe damage to more than 100 tree species in Taiwan [29]. Furthermore, the antifungal activity of diterpenoids, including ferruginol, taxodone, and taxodione, has been reported against two wood-decaying fungi, *Trametes versicolor* (white rot) and *Fomitopsis palustris* (brown rot), affecting coniferous species [30].

### 2.3. Structure–Activity Relationship Analysis

**Figure 6:**
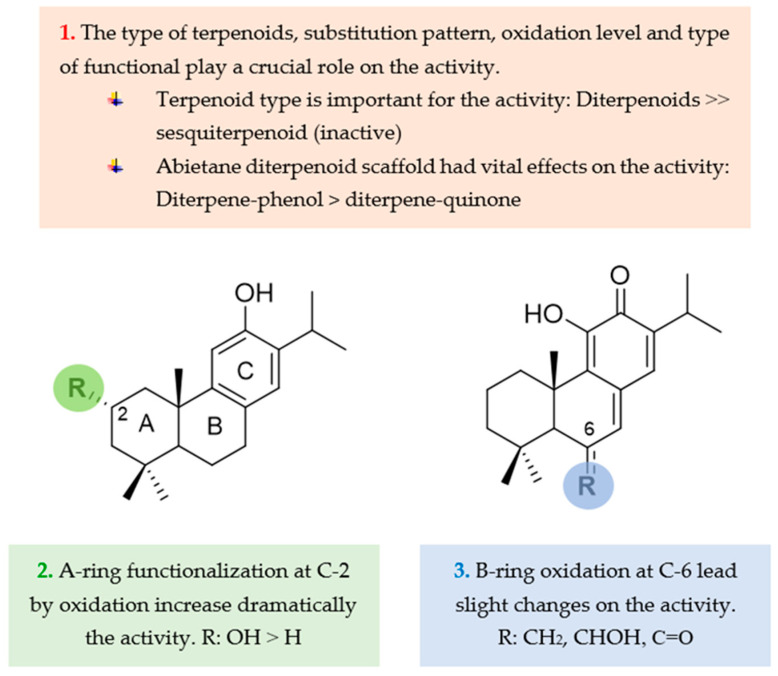
Structure–activity relationship (SAR) analysis of isolated compounds from cultivated *Salvia canariensis* as potential biofungicides.

Taking into consideration the %GI values (Table 2) and the effects of the oxidation pattern in the diterpenoid scaffold on the fungicidal activity, some structure–activity requirements can be established (Figure 6): (a) the secondary aliphatic alcohol seems to be an essential functional group for biological activity, and, in this sense, the hydroxylation at C-3 caused a notable increase in activity against the three phytopathogenic fungi (**1** vs. **5**); (b) the comparison of the antifungal activity of the abietane-type quinone diterpenoids and diterpene phenol (**3**, **4** and **5** vs. **2**) suggests that the oxidation of the phenolic skeleton to quinone had a detrimental effect on the activity; (c) the structural modification of the B-ring by oxidation at the C-7 position only leads to slight changes in activity (**2** vs. **3** vs. **4**).

### 2.4. Phytochemical Comparative Analysis

**Figure 7:**
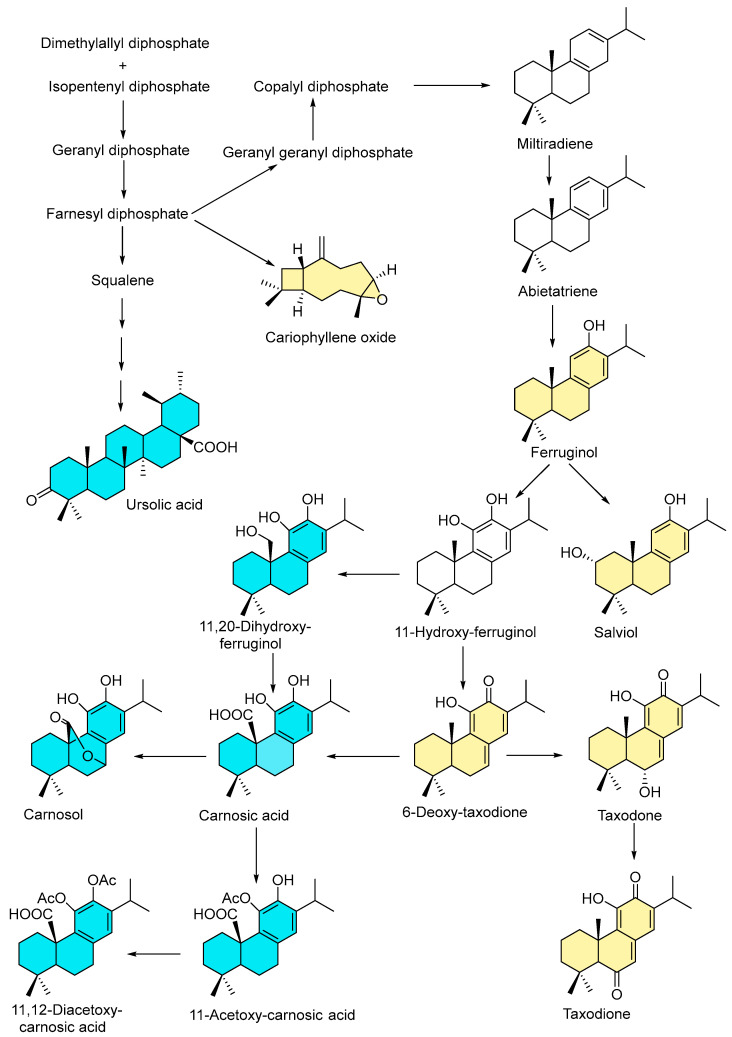
Overview of proposed biosynthetic network of abietane-type diterpenoids isolated from cultivated and wild-type *S. canariensis* by fungicidal bioguided fractionation. The color code for the isolated compounds is as follows: yellow is for terpenoids from cultivated plants and blue denotes compounds isolated from wild-type plants.

The present work was intended to validate the previously bioguided fractionation of wild-type *S. canariensis* as a potential source of biopesticides [17]. Along these lines, the comparative analysis revealed similar biopesticide profiles for the extract and fractions. However, distinctive phytochemical profiles of the isolated terpenoids between the cultivated and wild-type *S. canariensis* (Figure 7) were found. The analysis showed the following trends: (a) phytochemical studies revealed the presence of a sesquiterpenoid (caryophyllene oxide) and diterpenoids for cultivated plants and the combination of diterpenoid and triterpenoid (ursolic acid) compounds in the wild-type plant; (b) diterpenoid biosynthesis in the wild plant mainly yielded 11,12-orto-catechol-type diterpenoids functionalized at C-20 (carnosol, carnosic acid, 11,20-dihydroxyferruginol, 11-acetoxy carnosic acid, 11,12-diacetoxy carnosic acid), while cultivated plant biosynthesis produced quinone diterpenes (**2**–**4**) and phenolic diterpenes (**1** and **5**). (c) Moreover, the diterpenoid biosynthesis of cultivated plants presented a significant difference due to the unusual hydroxylation at the C-2 position as compared to the wild plant. (d) Overall, no metabolite has been isolated from the bioguided fractionation of cultivated or wild-type plants.

This untargeted comparative analysis reveals that these natural products are biosynthesized through interconnected routes or through a common pathway that diverges to yield different terpenoids. The diversity in the terpenoid content and oxygenation patterns of the diterpene scaffold offers an example of how plant cultivation can help to analyze the flexibility of plant metabolic pathways that enable them to change the terpene biosynthetic route rapidly and dynamically to adapt to the growing conditions. These distinguishing oxygenation patterns of the diterpene scaffold suggest that at least two independent abietane biosynthetic pathways may be involved in the phytochemical biosynthesis of cultivated and wild-type *S. canariensis*. Moreover, a comprehensive analysis of these results indicates that the environment plays a crucial role in the chemical profile of this plant.

Overall, the comparison of the antifungal profiles of the EtOH extract and fractions from the liquid–liquid partition of cultivated *S. canariensis* with that of the wild plant [17] against the three strains tested revealed similar fungicidal potency. In addition, the fungicidal activity of the most active compounds, the 11,12-diacetoxy carnosic acid isolated from the wild plant and the salviol diterpenoid (**5**) from the cultivated plant, revealed a similar biopesticide profile. These results are of relevance since they show that biodiversity can be preserved and yet sufficient plant mass can be obtained to improve plant production.

## 3. Materials and Methods

### 3.1. General

Optical rotations were measured on a Perkin Elmer 241 automatic polarimeter in CHCl_3_ at 25 °C and the [α]^D^ values are given in 10^−1^ deg cm^2^/g. The zero point of the polarimeter was applied with chloroform (1 mL). Compounds **1** (11.1 mg), **2** (15.6 mg), **3** (17.5 mg,) **4** (2.25 mg), **5** (5.3 mg), and **6** (9.4 mg) were dissolved in chloroform (1 mL). ^1^H NMR and ^13^C NMR were recorded at 300 K on Bruker Avance 500 and 600 NMR spectrometers (Karlsruhe, Germany). The spectra of compounds **1** (11.1 mg), **2** (15.6 mg), **3** (17.5 mg), **4** (2.25 mg), **5** (5.3 mg), and **6** (9.4 mg) were recorded in 0.5 mL of deuterated chloroform (CDCl_3_) or deuterated methanol (CD_3_OD). The spectra were obtained in NMR tubes with a 5 mm outer diameter. The chemical shifts (δ) were expressed in parts per million (ppm) with TMS as an internal reference and the coupling constants (*J*) in Hertz (Hz). Deuterated chloroform (δ_H_ 7.26, δ_C_ 77.0) was used as an internal reference. For the ^1^H NMR experiments, the relaxation delay was a 90° pulse, with a spectral width of 5500 Hz and 32 k data points. The Gaussian function was applied to enhance the spectral resolution, using −0.4 and 0.9 for Lorentzian broadening and Gaussian broadening, respectively. For the ^13^C NMR experiments, the corresponding parameters were a 30° pulse, 21,000 Hz, 62 k data points, and a 3.0 s relaxation delay. Silica gel 60 (particle size 15–40 and 63–200 μm, Macherey-Nagel) and Sephadex LH-20 (Pharmacia Biotech) were used for column chromatography, and silica gel 60 F254 (Macherey-Nagel) was used for analytical or preparative TLC. The spots were visualized by UV light and by heating silica gel plates sprayed with H_2_O-H_2_SO_4_-AcOH (1:4:20). All solvents used were of analytical grade and purchased from Panreac (Barcelona, Spain). The potato dextrose agar culture medium (PGA, Sigma-Aldrich, Madrid, Spain) and 9-cm-diameter Petri dishes (Sarstedt, Nümbrecht, Germany) were used for the maintenance of the fungal colonies and to carry out the bioassays. Tetracycline (50 mg/L, Sigma-Aldrich, Madrid, Spain) was added to avoid bacterial growth on the Petri dishes. Stock solutions of the tested samples were prepared with absolute ethanol (Sigma-Aldrich, Madrid, Spain). Fosbel-Plus (35% Fosetil Al and 35% Mancozeb) from Probelte S.A. (Murcia, Spain) was purchased in a phytosanitary product store and used as a reference fungicide for the tests against *A. alternata*, *B. cinerea*, and *F. oxysporum*. In the case of *B. cinerea*, Ortiva PC (Azoxystrobin 250 g/L, 22.8% p/p) from Syngenta España S.A (Madrid, Spain) was also used as a reference fungicide. Ethanol was used as a negative control, using one dish per pathogen and eight discs for each control.

### 3.2. Plant Cultivation, Seedling Production, and Plant Collection

The seeds of the species *Salvia canariensis* L. were collected from the natural environment and native plants belonging to the garden center of native flora at the Environmental Centre La Tahonilla (Cabildo Insular de Tenerife, Canary Island, Spain), situated at La Laguna (36°17′25″ N and 59°35′45″ E; 985 m above sea level). The conditions in the greenhouse during the sowing phase were 75 ± 2% humidity and 23 ± 2 °C. This material was cultivated using substrate compost (25%), coconut fiber (50%), and perlite (25%) and irrigated three times a week. The seedlings with 3–4 leaves were further transplanted into an individual pot. In this first phase of growth, the plants received only water to maintain the optimal conditions, because the application of fungicides can affect the roots’ development. At the potting phase, a solid fertilizer (8 gr/each pot, 16N-9P-12K) was added once a month to enhance the blooming. Once they were transplanted into a new pot, they were placed in the experimental field in May 2022 (325 plants) to grow until they were collected. The leaves of cultivated *S. canariensis* were gathered in November 2022. The leaves were spread on a tray, turned over occasionally, and air-dried at room temperature (22 ± 4 °C) for two weeks. The dried plant materials were ground and stored until extraction.

### 3.3. Workflow Procedure

#### 3.3.1. Plant Extract Preparation and Liquid–Liquid Partition Procedure

The air-dried and powdered leaves of cultivated *S. canariensis* (400.0 g) were extracted by maceration three times with 4 L of 96% ethanol (EtOH) for 24 h at room temperature (22 ± 4 °C). The extract was filtered, and the solvent was removed under reduced pressure at 40 °C on a rotary evaporator, yielding 51.7 g of crude extract. An aliquot (30.0 mg) of the extract was assayed on phytopathogenic fungi (*Fusarium oxysporum*, *Botrytis cinerea*, and *Alternaria alternata*), exhibiting remarkable fungicidal inhibition. The extract was subjected to further fractionation by liquid–liquid partition. Thus, the extract was sequentially fractionated using solvents of increasing polarity. Briefly, the ethanolic extract (51.7 g) was suspended in 500 mL of distilled water and successively extracted three times with 500 mL of hexane (Hx) and three times with 500 mL of ethyl acetate (EtOAc). The organic phases were concentrated under reduced pressure at 40 °C on a rotary evaporator to give the Hx (8.7 g) and EtOAc (22.3 g) fractions, whereas the aqueous residue was lyophilized, providing the aqueous fraction (H_2_O, 18.5 g). Aliquots (30.0 mg) of the three fractions from the liquid–liquid protocol were assayed for their antifungal activity, revealing that the hexane fraction was the most effective one (Table 1).

#### 3.3.2. Bioactivity-Guided Chromatographic Fractionation and Isolation

The hexane fraction (8.7 g) was subjected to silica gel column chromatography, using mixtures of hexane–EtOAc of increasing polarity (10:0 to 0:10) as the eluent to afford sixteen sub-fractions, which were combined based on their TLC profiles in sub-fractions A1–A13. The fungicidal activity analysis revealed that sub-fractions A3–A7, A11, and A12 were active against the tested phytopathogenic fungi, and they were subjected to several chromatography steps until the pure metabolites were obtained (Figure 1). In summary, each active sub-fraction was fractioned by polarity chromatography on silica gel (hexane–EtOAc, hexane–DCM, or DCM–EtOAc), affording around thirty sub-fractions, which were combined based on their TLC profiles. Finally, the sub-fractions were purified by size exclusion chromatography on a Sephadex LH-20 (isocratic mixtures of hexane–CHCl_3_–MeOH, 2:1:1 or CHCl_3_–MeOH, 1:1) or on silica gel (mixtures of increasing polarity: hexane–EtOAc, hexane–DCM, or DCM–EtOAc), or by preparative TLC (isocratic mixtures of hexane–EtOAc, hexane–DCM, or DCM–EtOAc), to give the pure compounds, identified as ferruginol (**1**) [20], 11-hydroxy-12-oxo-7,9 (11)-13-abietatriene (6-deoxy-taxodione, **2**) [21], taxodone (**3**) [22], taxodione (**4**) [23], salviol (**5**) [24], and caryophyllene oxide (**6**) [25] (Supplementary Materials, Experimental Part S1). The structural characterization of each isolated compound was carried out via the analysis of their NMR spectroscopic and spectrometric data (Supplementary Materials, Figures S1–S12) and the comparison of their spectral data with those reported in the literature.

### 3.4. Biological Assays

#### 3.4.1. Fungal Culture

The phytopathogenic fungi *Alternaria alternata*, *Botrytis cinerea*, and *Fusarium oxysporum* were maintained at 25 °C in darkness and periodically replicated in Petri dishes with PGA culture medium and tetracycline to avoid contamination. Strains of *B. cinerea* (B05.10) and *A. alternata* (Aa 100) were isolated from *Vitis vinifera* and *Lycopersicon esculentum*, respectively, both supplied by the Universidad de La Laguna, Tenerife. The *F. oxysporum f.* sp. *lycopersici* (2715) strain, isolated from *Lycopersicon esculentum*, was provided by the Colección Española de Cultivos Tipo (CECT) from Valencia, Spain.

#### 3.4.2. In Vitro Test Assay on Mycelium

The antifungal activity of the extract, fractions, sub-fractions, and isolated compounds was performed on the mycelium stages of *F. oxysporum*, *B. cinerea*, and *A. alternata* using an agar dilution protocol as previously described [19]. Briefly, an aliquot of each fraction/pure compound, dissolved in EtOH (50 mg/mL stock solution), was dissolved in warm PGA (final volume 5 mL) and placed in a 9 cm Petri dish. The medium containing the sample was allowed to solidify. Eight 4-mm-diameter discs of each fungus were deposited in Petri dishes with a 1 mg/mL final concentration of each tested sample or control and incubated for 48 h (*B. cinerea*) or 72 h (*A. alternata* and *F. oxysporum*). A Petri dish with eight discs was analyzed for each pathogen. Colony growth was measured with image-processing software, ImageJ 1.53e-Wayne Rasband. The diameter of each colony was measured twice, creating a cross, and the average of both measurements was taken. The percentage growth inhibition (%GI) was calculated using the equation %GI = [(C − T)/C] × 100, where C and T are the diameters of the control and test treated colonies, respectively. Fractions and sub-fractions exhibiting a %GI higher than 20% at 1 mg/mL were also evaluated at 0.5 and 0.1 mg/mL doses. The pure compounds’ growth inhibition assays were conducted at 0.1, 0.05, and 0.01 mg/mL. Fosbel-Plus and Ortiva PC were used as positive controls and EtOH was used as a negative control, using a dish per pathogen and eight discs for each control.

The concentration–response data were subjected to a Probit analysis to determine the EC_50_ (half maximal effective concentration) using the SPSS v28.0.1.1 software program (Chicago, IL, USA). EC_50_ values whose 95% confidence intervals did not overlap were considered statistically different.

#### 3.4.3. Statistical Analysis

All data are presented as the mean ± standard deviation (SD). Prior to analysis, the data were assessed for normality and homogeneity of variances using the Shapiro–Wilk and Levene tests, respectively, to ensure the validity of the ANOVA assumptions. Outliers were identified and evaluated individually to assess their impacts on the results, with any influential outliers excluded from the final analysis. The percentage of inhibition was analyzed using one-way analysis of variance (ANOVA) [31], and significant differences between the concentrations and treatments were further evaluated using Tukey’s honestly significant difference (HSD) test for multiple comparisons. A Probit analysis was also applied to assess the dose–response relationships where applicable. Results with a *p*-value < 0.05 were considered statistically significant. All analyses were performed using the Social Science Statistics software (2018 version).

## 4. Conclusions

Our previous work identified the potential of wild *S. canariensis*, and this new study overcame significant cultivation challenges and confirmed the preservation of the fungicidal properties in cultivated plants. This represents a crucial step in the long-term development of effective and sustainable biopesticides. Thus, the current research supports the notion that cultivated *S. canariensis* is a rich source of promising biofungicides against high-risk phytopathogens, offering a sustainable alternative that could maintain the conservation status and/or mitigate wild collection pressure. The bioassay-guided fractionation of the ethanolic extract successfully identified active diterpenoids, notably salviol, which could be considered a potential biopesticide candidate, exhibiting an outstanding antifungal profile, comparable to that of the commercial pesticides used as positive controls. The comparative analysis of the phytochemical composition between the wild-type and cultivated one revealed that the terpenoid composition depends on the environmental growing conditions. These findings provide valuable insights for the future application of cultivated *S. canariensis* and highlight the potential of abietane-type diterpenoids as promising biopesticides against high-risk phytopathogens. Nonetheless, further studies are warranted to elucidate their modes of action and optimize the extraction process for their application in sustainable crop protection.

## Figures and Tables

**Table 1:** Antifungal effects (% growth inhibition, %GI) of the ethanolic extract and liquid–liquid fractions from cultivated *S. canariensis* against *Alternaria alternata*, *Botrytis cinerea*, and *Fusarium oxysporum*.

Sample	*Alternaria alternata*			*Botrytis cinerea*			*Fusarium oxysporum*		
	1 mg/mL	0.5 mg/mL	0.1 mg/mL	1 mg/mL	0.5 mg/mL	0.1 mg/mL	1 mg/mL	0.5 mg/mL	0.1 mg/mL
E-EtOH	68.7 ± 3.7	62.1 ± 1.6	45.0 ± 3.3	41.1 ± 3.3	38.3 ± 6.9	29.5 ± 6.6	53.6 ± 0.8	45.9 ± 2.8	21.3 ± 2.4
F-Hx	73.5 ± 1.3	67.3 ± 1.5	44.3 ± 3.2	66.6 ± 2.9	55.7 ± 4.8	19.3 ± 4.0	52.4 ± 1.6	46.7 ± 2.2	26.6 ± 2.5
F-EtOAc	59.3 ± 1.4	55.9 ± 1.9	46.1 ± 0.7	19.2 ± 5.6	ND	ND	57.1 ± 1.5	55.5 ± 3.4	30.4 ± 1.1
F-H_2_O	46.7 ± 2.8	39.2 ± 2.3	27.0 ± 3.2	NA	ND	ND	41.2 ± 2.9	36.9 ± 2.5	26.4 ± 1.8
C	93.5 ± 3.6	87.8 ± 5.5	74.2 ± 4.8	83.3 ± 3.0	73.5 ± 5.8	23.6 ± 4.8	93.6 ± 4.8	92.6 ± 6.5	78.8 ± 4.7

%GI: The values are expressed as the percentage of growth inhibition relative to the control growth in the absence of inhibitory agents (negative control). E-EtOH: ethanolic extract; F-Hx: hexane fraction; F-EtOAc: ethyl acetate fraction; F-H_2_O: water fraction. NA: non-active (%GI ˂ 10%). ND: not determined. C: Fosbel-Plus was used as a positive control. The data shown are the averages of eight independent experiments ± SD (standard deviation).

**Table 2:** Antifungal effects (% growth inhibition) of compounds **1**–**6** isolated from cultivated *Salvia canariensis* leaves against *Alternaria alternata*, *Botrytis cinerea*, and *Fusarium oxysporum*.

Sample	*A. alternata*			*B. cinerea*			*F. oxysporum*		
	0.1 mg/mL	0.05 mg/mL	0.01 mg/mL	0.1 mg/mL	0.05 mg/mL	0.01 mg/mL	0.1 mg/mL	0.05 mg/mL	0.01 mg/mL
1	35.3 ± 3.5	33.1 ± 2.9	21.9 ± 4.5	37.1 ± 6.8	17.9 ± 4.0	21.4 ± 4.8	23.5 ± 3.1	20.4 ± 2.5	14.6 ± 3.0
2	40.9 ± 3.0	35.6 ± 3.9	NA	49.2 ± 6.1	23.1 ± 4.4	NA	23.9 ± 5.5	NA	ND
3	39.2 ± 3.3	39.1 ± 6.5	25.6 ± 3.9	60.3 ± 5.4	46.3 ± 6.3	NA	27.8 ± 6.8	17.2 ± 3.7	ND
4	40.4 ± 4.0	36.1 ± 3.5	28.6 ± 3.1	45.4 ± 7.2	38.4 ± 4.7	NA	33.7 ± 4.1	21.9 ± 6.0	ND
5	72.3 ± 3.9	68.5 ± 2.7	43.3 ± 2.6	81.2 ± 3.9	71.7 ± 2.8	42.7 ± 4.4	74.1 ± 2.9	62.3 ± 2.7	46.6 ± 2.7
6	11.9 ± 4.6	ND	ND	NA	ND	ND	NA	ND	ND
C1	74.2 ± 4.8	65.5 ± 4.0	38.1 ± 3.8	23.6 ± 4.8	21.9 ± 3.8	13.5 ± 3.6	78.8 ± 4.7	44.4 ± 4.8	28.7 ± 2.5
C2	30.4 ± 2.1	31.2 ± 4.0	33.4 ± 1.9	67.8 ± 4.1	50.0 ± 7.3	32.6 ± 6.1	53.2 ± 5.1	51.6 ± 5.1	32.2 ± 6.2

The values are expressed as the percentage of growth inhibition (%GI) relative to the control growth in the absence of inhibitory agents (negative control). Compounds with growth inhibition higher than 20% at 0.1 mg/mL were assayed at lower concentrations (0.05 and 0.01 mg/mL). NA: non-active (% GI ˂ 10%). ND: not determined. C1: Fosbel-Plus and C2: Azoxytrobin were used as positive controls. The data shown are the averages of eight independent experiments ± SD (standard deviation).

## Data Availability

The data are contained within the article or Supplementary Materials.

## Supplementary Materials

The following supporting information can be downloaded at: https://www.mdpi.com/article/10.3390/ijms252413323/s1.
