# Supplementary material for: Bioassay-Guided Fractionation Networking for Discovery of Biofungicides from Cultivated Salvia canariensis
Source: Int J Mol Sci. 2024 Dec 12;25(24):13323. doi: 10.3390/ijms252413323 (PMC11677097; doi:10.3390/ijms252413323)
Supplement: Supplementary file 1 [file ijms-25-13323-s001.zip › ijms-3244252-supplementary.pdf]

Electronic Supporting Information

Bioassay-Guided Fractionation Networking for Discovery of Biofungicides from cultivated *Salvia canariensis*

Eduardo Hernández-Álvarez[1], Samuel Rodríguez Sabina[2], Guacimara González-Delgado[3], Carolina P. Reyes[4], Cristina Giménez[2], M. Ángeles Llaría-López[3], Raimundo Cabrera[2], Isabel L. Bazzochi[1], Ignacio A. Jiménez[1,*]

[1]*Instituto Universitario de Bio-Orgánica Antonio González, Departamento de Química Orgánica, Avenida Astrofísico Francisco Sánchez 2, 38206 La Laguna, Tenerife, Spain.*

[2]*Departamento de Botánica, Ecología y Fisiología Vegetal, Facultad de Ciencias, Sección Biología, Universidad de La Laguna, Avenida Astrofísico Francisco Sánchez, 38206 La Laguna, Tenerife, Spain.*

[3]*Área de Medio Natural y Seguridad, C/ Las Macetas s/n, Pabellón Insular Santiago Martín, 38108 La Laguna, Tenerife, Spain.*

[4]*Instituto Universitario de Bio-Orgánica Antonio González, Departamento de Bioquímica, Microbiología, Biología Celular y Genética, Universidad de La Laguna, Avenida Astrofísico Francisco Sánchez 2, 38206 La Laguna, Tenerife, Spain.*

**Correspondence: ignadiaz@ull.edu.es; Tel.: +34-922318594*

Table of Contents

Experimental Part S1. Bioguided fractionation**:** Extraction and Isolation.

Figures S1-S12. ^{1}H NMR, ^{13}C NMR and mass spectra of the known metabolites (**1-6**) isolated from cultivated *Salvia canariensis* leaves.

Table S1. Antifungal effects (% Growth Inhibition) of extract, fractions, and sub-fractions from cultivated *Salvia canariensis* leaves against *Alternaria alternata*, *Botrytis cinerea* and *Fusarium oxysporum.*

Bioguided fractionation: Extraction and Isolation

The air-dried and powdered leaves of cultivated *S. canariensis* (400.0 g) were extracted by maceration with 96% EtOH (4 L x 3 times) at room temperature for 24 h for each maceration process and concentrated under reduced pressure to yield 51.7 g of residue, which was assayed on phytopathogenic fungi (*Fusarium oxysporum*, *Botrytis cinerea*, and *Alternaria alternata*) giving a potent activity. Therefore, the ethanolic extract was suspended in water (H_2O) and solvent-solvent partitioned sequentially with hexanes (Hx) and ethyl acetate (EtOAc). The organic phases were concentrated under reduced pressure to give Hx (8.7 g) and EtOAc (22.3 g) fractions, whereas the aqueous residue was lyophilized providing the H_2O fraction (20.5 g). Biological evaluation revealed that the organic fractions were active against the phytopathogenic fungi and were further investigated. The most active fraction (Hx, 8.7 g) was chromatographed on a silica gel column, using mixtures of hexanes/EtOAc of increasing polarity (10:0 to 0:10) as eluent to afford sixteen sub-fractions, which were combined based on their TLC profile in sub-fractions A1-A13. Fungicidal activity revealed that sub-fractions A3-A7, A11 and A12 were active against the strain of phytopathogenic fungi and were subjected to several chromatography steps until obtaining the pure compounds. Sub-fraction A3 (3.87 g) was chromatographed on a silica gel column, using mixtures of hexanes/EtOAc of increasing polarity (10:0 to 8:2) as eluent to afford forty-eight sub-fractions, which were combined based on their TLC profile in sub-fractions A3A-A3K. Sub-fraction A3A (426.1 mg) was further purified on Sephadex LH-20 (hexanes/$CHCl_3$/MeOH, 2:1:1), affording sub-fractions five sub-fractions A3A1-A3A5, based on their TLC profile. Sub-fraction A3A4 (172.9 mg) was chromatographed on a silica gel column, using mixtures of hexanes/EtOAc of increasing polarity (10:0 to 9:1) as eluent to afford six sub-fractions A3A4A-A3A4F, based on their TLC profile. Sub-fraction A3A4A (17.2 mg) was further purified by preparative TLC

(hexanes/EtOAc, 8.5:1.5) to give compound **4** (taxodione, $[\alpha]^{20}_{D}$ +42.7, *c* 0.2, $CHCl_3$); 1.0 mg). Sub-fraction A3A4B (48.0 mg) was further purified by preparative TLC (hexanes/EtOAc, 8.5:1.5) to give compound **2** (6-deoxy-taxodione, $[\alpha]^{20}_{D}$ -27.1, *c* 1.6, $CHCl_3$); 10.6 mg). Sub-fraction A3E (191.5 mg) was chromatographed on a silica gel column, using mixtures of hexanes/DCM of increasing polarity (7:3 to 6:4) as eluent to afford three sub-fractions A3E1-A3E3, based on their TLC profile. Sub-fractions A3E1 (11.8 mg) and A3E3 (42.4 mg) were identified as compounds **1** (ferruginol, $[\alpha]^{20}_{D}$ +42.3, *c* 1.1, $CHCl_3$) and **6** (caryophyllene oxide, $[\alpha]^{20}_{D}$ +1.5, *c* 0.9, $CHCl_3$), respectively. Sub-fraction A3F (173.3 mg) was chromatographed on a silica gel column, using mixtures of hexanes/DCM of increasing polarity (8:2 to 4:6) as eluent to afford five sub-fractions A3F1-A3F5, based on their TLC profile. Sub-fractions A3F1 (13.8 mg) and A3E4 (2.3 mg) were identified as compounds **1** ferruginol and **6** caryophyllene oxide, respectively.

Sub-fraction A4 (856.0 mg) was chromatographed on a silica gel column, using mixtures of hexanes/DCM of increasing polarity (1:1 to 1:9) as eluent to afford forty-one sub-fractions, which were combined based on their TLC profile in sub-fractions A4A-A4I. Sub-fraction A4A (86.0 mg) was chromatographed on a silica gel column, using mixtures of hexanes/EtOAc of increasing polarity (10:0.1 to 10:0.5) as eluent to afford three sub-fractions A4A1-A4A3, based on their TLC profile. Sub-fractions A4A1 (14.1 mg) and A4A3 (16.6 mg) were identified as compound 6-deoxy-taxodione (**2**) and taxodione (**4**), respectively. Sub-fraction A4C (17.9 mg) was further purified by preparative TLC (hexanes/diethyl ether 5:5) to give compounds **3** (taxodone, $[\alpha]^{20}_{D}$ +17.0, *c* 1.8, $CHCl_3$; 9.3 mg). Sub-fraction A5 (612.2 mg) was chromatographed on a silica gel column, using mixtures of hexanes/EtOAc of increasing polarity (10:0 to 7:3) as eluent to afford ninety-seven sub-fractions, which were combined based on their TLC profile in sub-fractions A5A-A5G. Sub-fractions A5A (0.9 mg) and A5C (7.5 mg) were identified as compound 6-deoxy-taxodione (**2**)

and taxodione (**4**), respectively. Sub-fraction A6 (667.7 mg) was chromatographed on a silica gel column, using mixtures of hexanes/EtOAc of increasing polarity (8:2 to 4:6) as eluent to afford thirty-five sub-fractions, which were combined based on their TLC profile in sub-fractions A6A-A6F. Sub-fractions A6A (38.2 mg) and A6C (92.0 mg) were identified as compound taxodione (**4**) and taxodone (**3**), respectively.

Sub-fraction A7 (312.1 mg) was chromatographed on a silica gel column, using mixtures of hexanes/EtOAc of increasing polarity (8:2 to 4:6) as eluent to afford twenty-seven sub-fractions, which were combined based on their TLC profile in sub-fractions A7A-A7D. Sub-fraction A7A (17.2 mg) was identified as compound taxodione (**4**). Sub-fraction A7C (105.0 mg) was further purified on Sephadex LH-20 (hexanes/$CHCl_3$/MeOH, 2:1:1), affording sub-fractions A7C1-A7C10. Sub-fractions A7C4 (4.5 mg) and A7C6 (14.2 mg) were identified as compound taxodione (**4**) and taxodone (**3**), respectively. Sub-fraction A11 (341.7 mg) was chromatographed on a silica gel column, using mixtures of dichloromethane (DCM)/acetone of increasing polarity (9:1 to 6:4) as eluent to afford a hundred sub-fractions, which were combined based on their TLC profile in sub-fractions A11A-A11D. Sub-fraction A11D (122.0 mg) was further purified by Sephadex LH-20 column chromatography ($CHCl_3$/MeOH, 1:1), affording sub-fractions A11D1-A11D4. Sub-fraction A11D (38.2 mg) was further purified by preparative TLC (hexanes/diethyl ether 5:5) to give compounds **5** (salviol, $[\alpha]^{20}_{D}$ +25.7, *c* 0.5, $CHCl_3$, 9.4 mg). Sub-fraction A12 (119.8 mg) was chromatographed on a silica gel column, using mixtures of DCM/acetone of increasing polarity (10:0 to 6:4) as eluent to afford seven sub-fractions A12A-A12G. Sub-fraction A12F (10.6 mg) was further purified by Sephadex LH-20 column chromatography ($CHCl_3$/MeOH, 1:1), affording sub-fractions A12F1-A12F2. Sub-fractions A12F2 (6.2 mg) was identified as compound **5**, salviol.

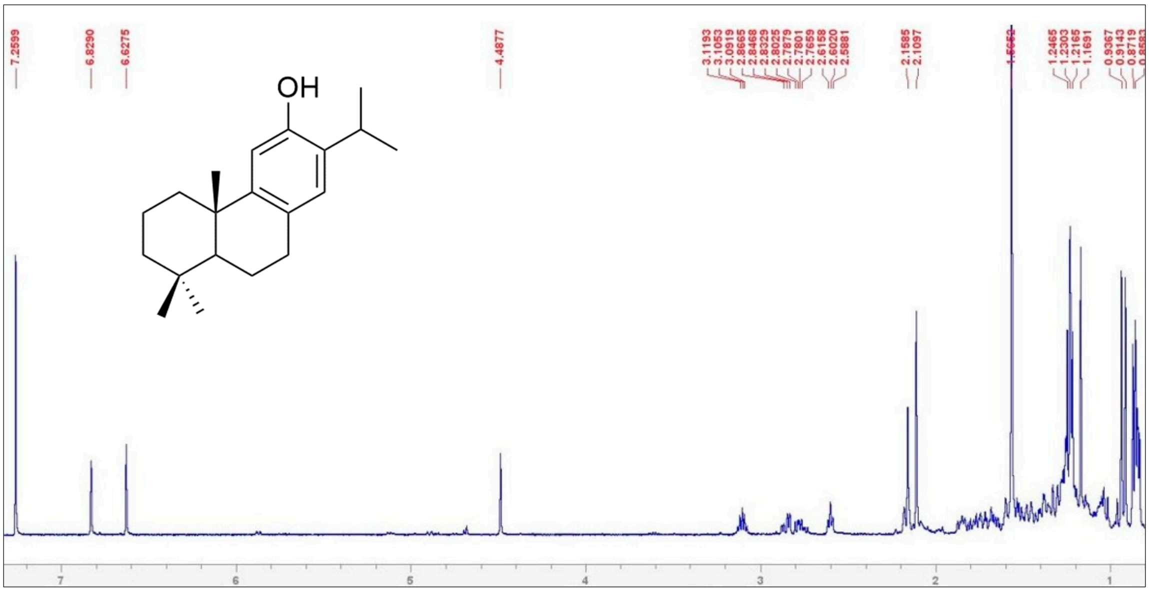

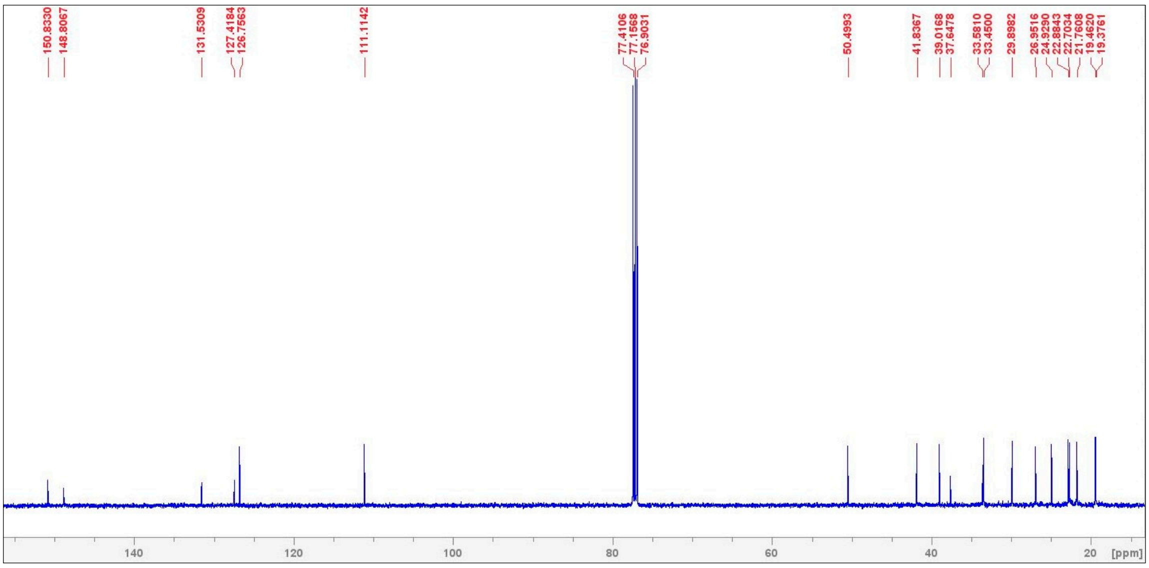

Figure S1. ^{1}H and ^{13}C NMR spectra of **1** (ferruginol) in $CDCl_3$ (500 and 125 MHz).

Monoisotopic Mass, Even Electron Ions
100 formula(e) evaluated with 1 results within limits (all results (up to 1000) for each mass)
Elements Used:
C: 0-60 H: 0-80 O: 0-10 Na: 0-2
20240604_FERR 79 (2.695)
2: TOF MS ES-

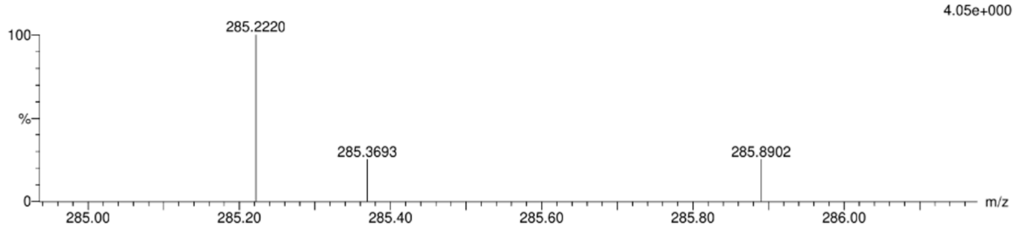

Minimum:	30.00				-1.5			
Maximum:	100.00		5.0	5.0	200.0			
Mass	RA	Calc. Mass	mDa	PPM	DBE	i-FIT	i-FIT (Norm)	Formula
285.2220	100.00	285.2218	0.2	0.7	6.5	15.8	0.0	C20 H29 O

Figure S2. Mass spectrum of compound **1** (ferruginol)

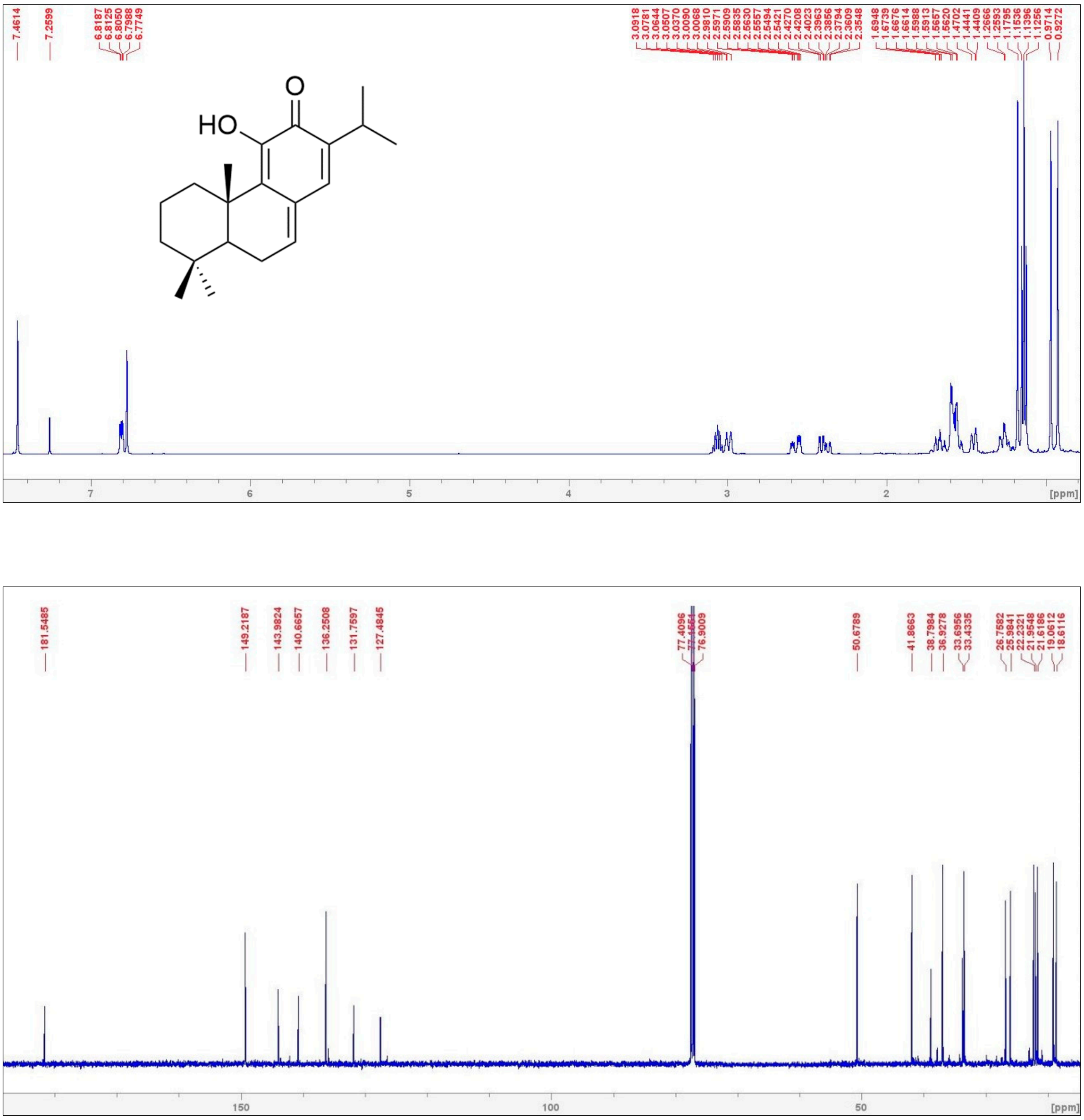

Figure S3. ^{1}H and ^{13}C NMR spectra of **2** (6-deoxy-taxodione) in $CDCl_3$ (500 and 125 MHz).

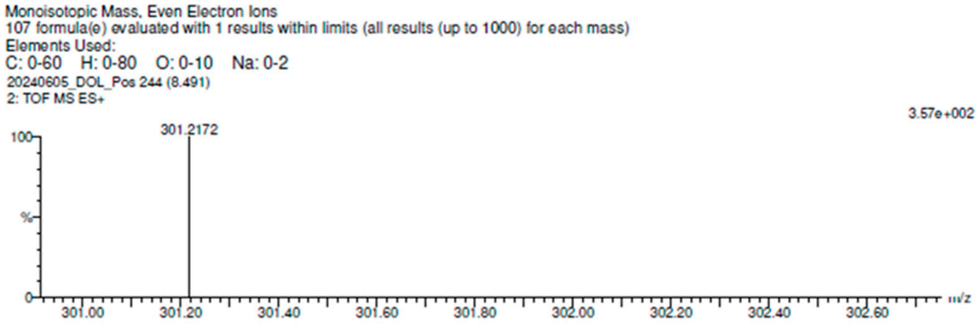

Minimum:	30.00				-1.5			
Maximum:	100.00		5.0	5.0	200.0			
Mass	RA	Calc. Mass	mDa	PPM	DBE	i-FIT	i-FIT (Norm)	Formula
301.2172	100.00	301.2168	0.4	1.3	6.5	68.8	0.0	C20 H29 O2

Figure S4. Mass spectrum of compound **2** (6-deoxy-taxodione)

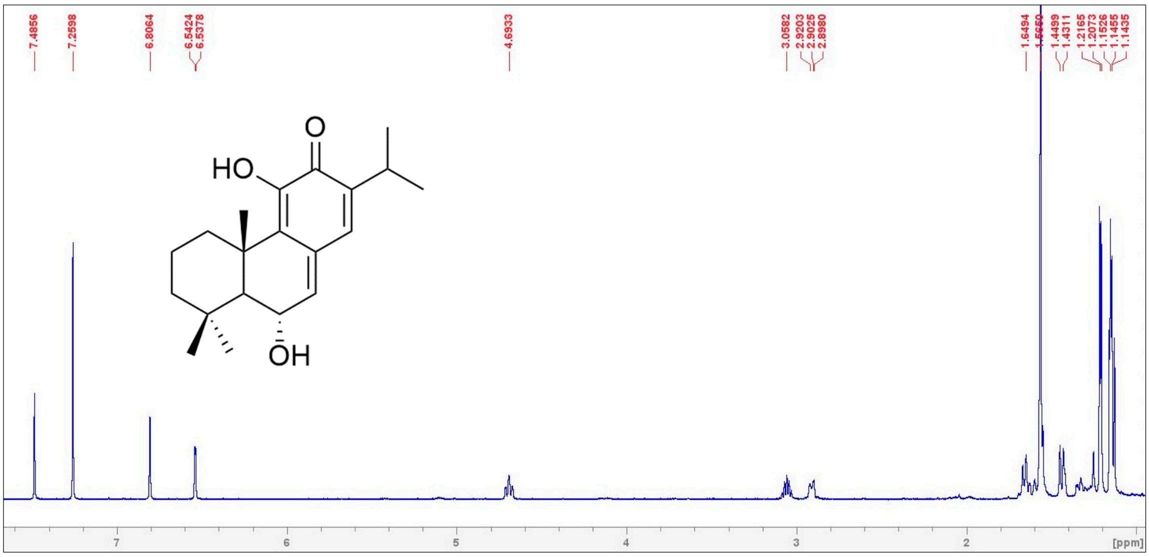

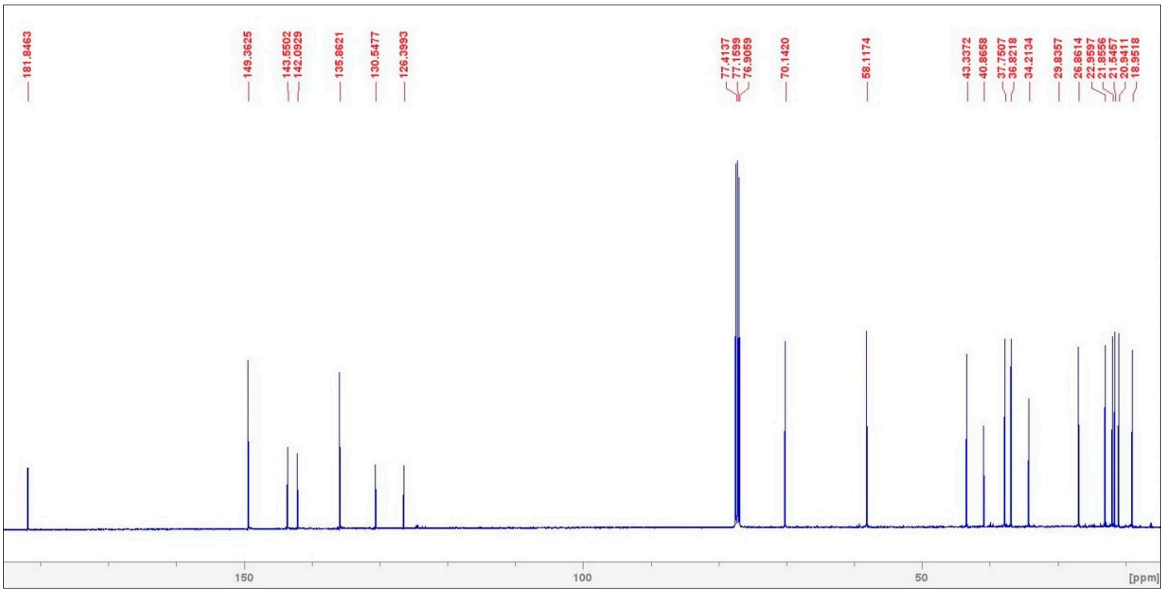

Figure S5. ^{1}H and ^{13}C NMR spectra of **3** (taxodone) in $CDCl_3$ (500 and 125 MHz).

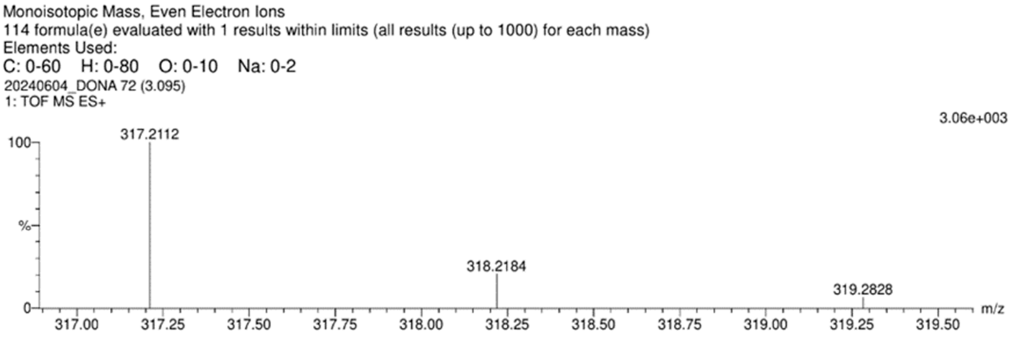

Minimum:	30.00				-1.5				
Maximum:	100.00		5.0	5.0	200.0				
Mass	RA	Calc. Mass	mDa	PPM	DBE	i-FIT	i-FIT (Norm)	Formula
317.2112	100.00	317.2117	-0.5	-1.6	6.5	25.0	0.0	C20 H29 O3

Figure S6. Mass spectrum of compound **3** (taxodone)

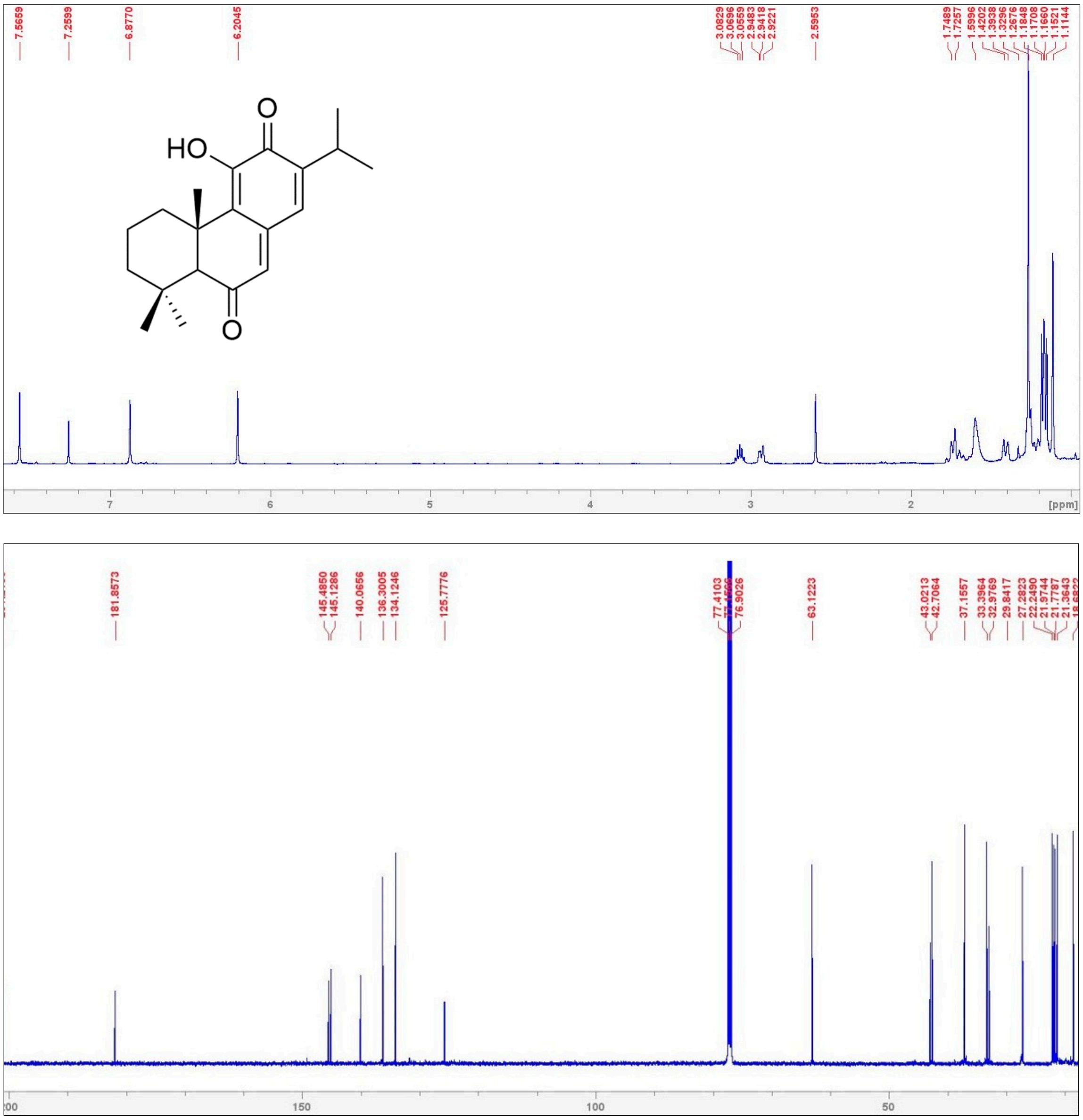

Figure S7. ^{1}H and ^{13}C NMR spectra of **4** (taxodione) in $CDCl_3$ (500 and 125 MHz).

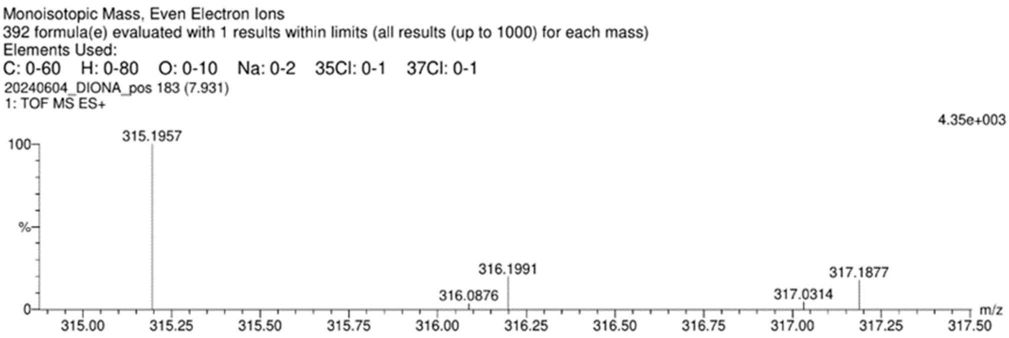

Minimum:	30.00				-1.5			
Maximum:	100.00		5.0	5.0	200.0			
Mass	RA	Calc. Mass	mDa	PPM	DBE	i-FIT	i-FIT (Norm)	Formula
315.1957	100.00	315.1960	-0.3	-1.0	7.5	46.3	0.0	C20 H27 O3

Figure S8. Mass spectrum of compound **4** (taxodione)

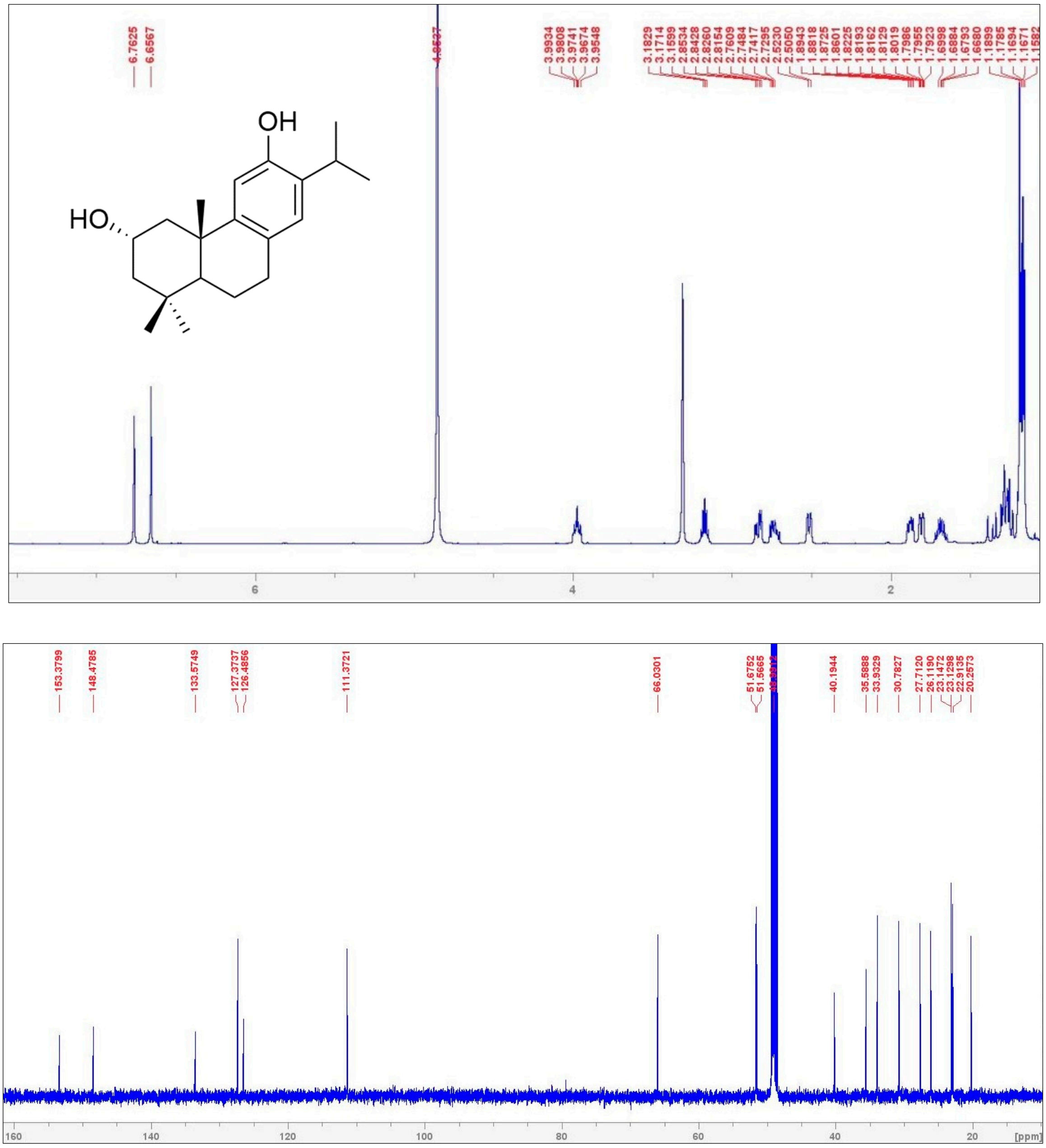

Figure S9. ^{1}H and ^{13}C NMR spectra of **5** (salviol) in CD_3OD (600 and 150 MHz).

Monoisotopic Mass, Even Electron Ions

510 formula(e) evaluated with 1 results within limits (all results (up to 1000) for each mass)

Elements Used:

C: 0-30 H: 0-60 O: 0-10 Na: 0-1

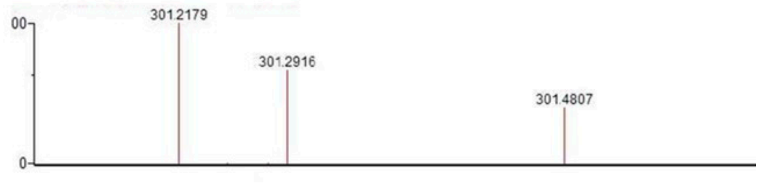

Minimum:	20.00			-1.5						
Maximum:	100.00	5.0	5.0	250.0						
Mass	RA	Calc. Mass	mDa	PPM	DBE	i-FIT	Norm	Conf(%)	Formula
301.2179	100.00	301.2168	1.1	3.7	6.5	74.7	n/a	n/a	C20 H29 O2

Figure S10. Mass spectrum of compound **5** (salviol)

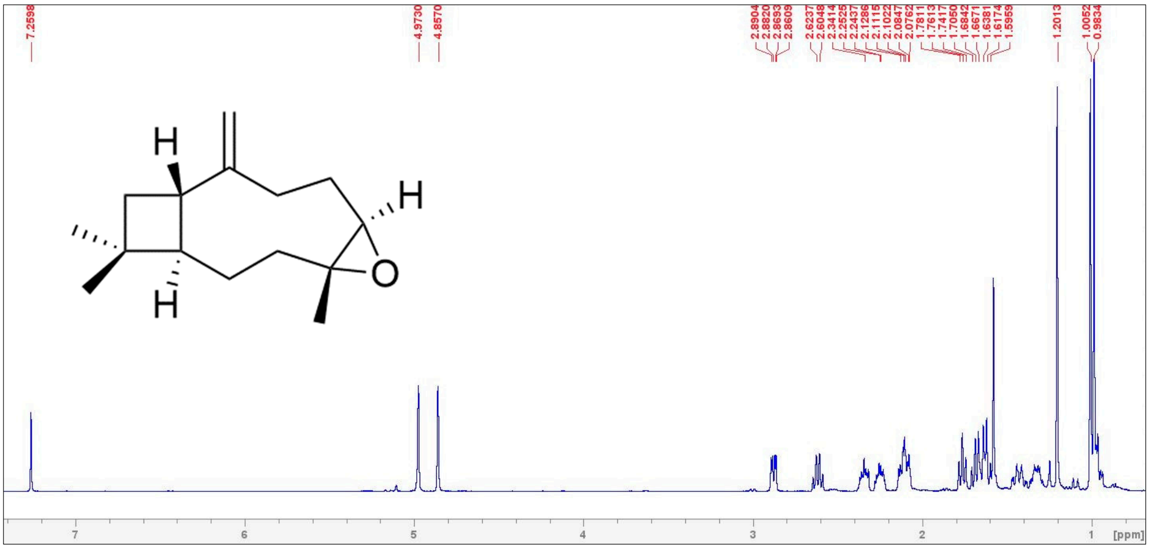

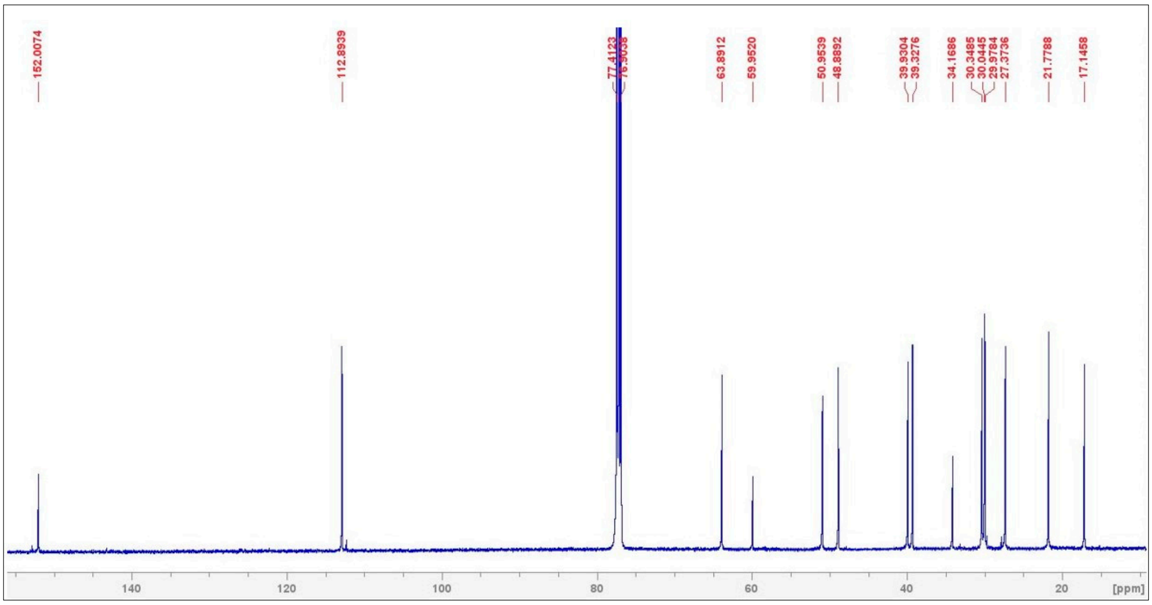

Figure S11. ^{1}H and ^{13}C NMR spectra of **6** (cariophyllene oxide) in $CDCl_3$ (500 and 125 MHz).

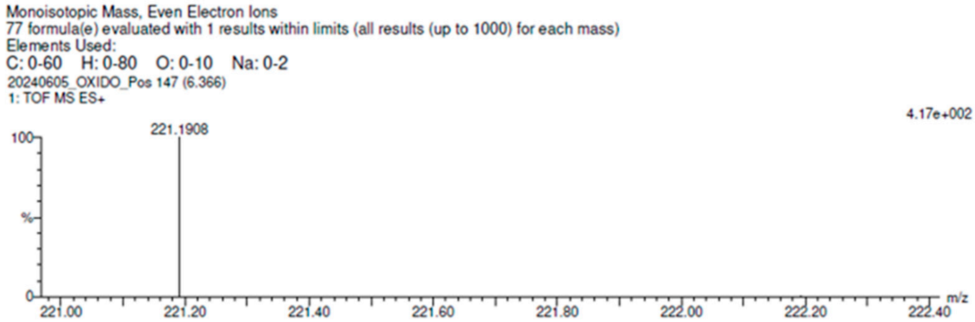

Minimum:	30.00				-1.5			
Maximum:	100.00		5.0	5.0	200.0			
Mass	RA	Calc. Mass	mDa	PPM	DBE	i-FIT	i-FIT (Norm)	Formula
221.1908	100.00	221.1905	0.3	1.4	3.5	12.2	0.0	C15 H25 O

Figure S12. Mass spectrum of compound **6** (cariophyllene oxide)

Table S1. Antifungal effects (% Growth Inhibition) of extract, fractions, and sub-fractions from leaves of cultivated *Salvia canariensis* against *Alternaria alternata*, *Botrytis cinerea* and *Fusarium oxysporum.*

**Sample**	***A. alternata***			***B. cinerea***			***F. oxysporum***		
	1 mg/mL	0.5 mg/ml	0.1 mg/ml	1 mg/mL	0.5 mg/ml	0.1 mg/ml	1 mg/mL	0.5 mg/ml	0.1 mg/ml
Extract EtOH	68.6 ± 3.7	62.1 ± 1.6	45.0 ± 3.3	41.1 ± 3.3	38.3 ± 6.9	29.5 ± 6.6	53.6 ± 0.8	45.9 ± 2.8	21.39 ± 2.4
Fraction Hx	73.5 ± 1.3	67.26 ± 1.5	44.35 ± 3.2	66.6 ± 2.9	55. 7 ± 4.8	19.3 ± 4.0	52.4 ± 1.6	46.7 ± 2.2	26.6 ± 2.5
A1	NA	ND	ND	NA	ND	ND	19.0 ± 1. 6	ND	ND
A2	19.7 ± 1.5	ND	ND	49.9 ± 5.5	45.7 ± 4.2	24.6 ± 5.5	27.9 ± 2.8	22.5 ± 3.4	NA
A3	68.9 ± 2.8	56.5 ± 2.3	14.6 ± 3.1	84.0 ± 4.4	80.3 ± 5.1	34.7 ± 5.0	55.2 ± 2.6	34.9 ± 2.7	NA
A4	65.9 ± 1.3	64.1 ± 1.4	49.9 ± 2.8	80.0 ± 4.9	77.9 ± 3.4	62.8 ± 5.4	57.1 ± 1.5	55.5 ± 3.4	30.4 ± 1.1
A5	60.6 ± 1.6	55.9 ± 1.1	27.4 ± 0.8	84.2 ± 4.5	80.0 ± 4.9	35.0 ± 4.3	41.2 ± 2.9	36.9 ± 2.5	26.4 ± 1.8
A6	60.8 ± 3.9	51.9 ± 3.0	37.6 ± 3.3	79.4 ± 2.7	79.2 ± 3.1	43.0 ± 3.7	47.2 ± 2.3	41.5 ± 3.2	27.3 ± 2.0
A7	62.1 ± 0.9	53.9 ± 1.2	45.7 ± 1.8	79.8 ± 4.1	82.4 ± 1.9	56.4 ± 6.1	58.6 ± 2.5	46.2 ± 2.8	45.5 ± 1.8
A8	55.2 ± 2.2	46.1 ± 2.0	43.3 ± 2.6	68.6 ± 3.8	66.7 ± 3.9	31.2 ± 5.5	48.5 ± 1.9	40.1 ± 2.0	36.6 ± 3.8
A9	49.9 ± 2.2	41.5 ± 1.3	23.4 ± 3.4	78.1 ± 4.0	59.7 ± 4.5	12.6 ± 3.6	39.2 ± 2.5	36.6 ± 2.7	21.0 ± 2.6
A10	51.2 ± 1.5	50.1 ± 2.8	34.2 ± 3.6	62.5 ± 5.2	50.6 ± 4.0	33.5 ± 3.5	45.9 ± 1.8	44.5 ± 1.5	26.6 ± 2.4
A11	73.2 ± 2.0	68.2 ± 2.6	61.9 ± 2.5	87.8 ± 2.5	86.4 ± 1.6	83.4 ± 2.7	85.9 ± 1.1	81.4 ± 0.9	69.7 ± 3.8
A12	65.4 ± 1.5	62.8 ± 1.5	47.7 ± 2.1	83.8 ± 2.8	73.4 ± 1.9	53.7 ± 4.0	73.4 ± 2.2	72.1 ± 2.7	51.2 ± 1.6
A13	53.4 ± 1.3	51.9 ± 1.3	39.5 ± 1.7	65.0 ± 3.6	49.9 ± 4.7	32.5 ± 4.1	46.4 ± 2.3	44.8 ± 1.9	30.7 ± 3.4
Fraction EtOAc	59.3 ± 1.4	55.9 ± 1.9	46.1 ± 0.7	19.2 ± 5.5	ND	ND	57.1 ± 1.5	55.5 ± 3.4	30.4 ± 1.1
Fraction H_2O	46.7 ± 2.8	39.2 ± 2.3	27.0 ± 3.2	NA	ND	ND	41.2 ± 2.9	36.9 ± 2.5	26.4 ± 1.8
Fosbel-Plus	93.5 ± 3.6	87.8 ± 5.5	74.2 ± 4.8	83.3 ± 3.0	73.5 ± 5.8	23.6 ± 4.8	93.4 ± 4.8	92.6 ± 6.5	78.8 ± 4.7

Extract/fractions/subfractions with an inhibition growth higher than 20% at 1 mg/mL were assayed at lower concentrations (0.5 and 0.1 mg/mL). % Growth Inhibition: Means ± standard deviation (SD). NA: not active (% inhibition ≤10); ND: not determined. Fosbel-Plus was used as a positive control.
